# The intrinsic link between the double burden of dental caries and stunting in children: the gut microbiota plays a key role

**DOI:** 10.1186/s12866-026-04804-3

**Published:** 2026-02-07

**Authors:** Wenqing Huang, Qiang Teng, Siyu Li, Jiaqi Bai, Yuxi Wang, Mengzhen Tang, Hongbing Chen, Jian Yang, Cuixiang Wan

**Affiliations:** 1 Department of Endodontics, Affiliated Stomatological Hospital, Nanchang University, 49, Fuzhou Road, Nanchang, 330006 Jiangxi China; 2 Jiangxi Province Key Laboratory of Oral Biomedicine, Nanchang, 330006 China; 3 Jiangxi Province Clinical Research Center for Oral Diseases, Nanchang, 330006 China; 4 State Key Laboratory of Food Science and Resources, Nanchang University, 235, Nanjing East Road, Nanchang, 330047 China; 5 Sino-German Joint Research Institute, Nanchang University, Nanchang, 330047 China; 6 College of Food Science and Technology, Nanchang University, Nanchang, 330047 China

**Keywords:** Dental caries, Stunting, Oral microbiota, Gut microbiota, Serum phospholipids

## Abstract

**Introduction:**

Currently, a significant number of children are experiencing the dual challenges of dental caries and malnutrition. This study aims to investigate the potential correlation between dental caries and malnutrition.

**Methods:**

Record the lifestyle variables, oral health conditions, and growth parameters of the subjects. Dental plaque and fecal samples were collected for 16 S rDNA sequencing to analyze the characteristics of the oral and gut microbiota. Furthermore, untargeted metabolomics was employed to investigate changes in the serum metabolome of patients with severe dental caries.

**Results:**

Poor lifestyle can alter the oral microbial composition, increasing the risk of developing dental caries. Changes in the oral microbiota of patients with dental caries affect the gut microbiota, resulting in an increased abundance of genera such as *Dialister*, *Megasphaera*, and *Agathobacter*. Sphingomyelin, phosphatidylcholine, lysophosphatidylcholine, lysophosphatidylethanolamine, and phosphatidylethanolamine exhibited a significant positive correlation with the height-for-age Z-score. In patients with severe dental caries, the serum concentrations of these phospholipids were notably reduced, which was closely related to changes in the gut microbiota.

**Conclusion:**

Dental caries disrupted the gut microbiota by altering the oral microbiota, leading to changes in the serum phospholipid profile of individuals with severe dental caries, ultimately raising the risk of stunting in children.

**Supplementary Information:**

The online version contains supplementary material available at 10.1186/s12866-026-04804-3.

## Introduction

Approximately 45% of the global population suffers from oral diseases, with untreated caries being the most prevalent disease worldwide [1]. Despite a recent decrease in the prevalence of dental caries, it remains the most significant oral health burden worldwide [2]. Currently, malnutrition is also a significant global health issue, including wasting, stunting, underweight, and overweight [3]. In 2022, 22.3% of children under the age of five experienced stunting, 6.8% with wasting, and 5.6% with overweight [4]. Lifestyle habits, maternal and child care, health services, and environmental factors are all potential causes of dental caries and malnutrition. Since dental caries and malnutrition share overlapping causative factors and affected populations, understanding the intrinsic connection between the two is crucial for preventing the occurrence of both dental caries and malnutrition.

Cariogenic microorganisms in the biofilm on the tooth surface have a close correlation to the onset of dental caries. *Streptococcus mutans* has long been recognized as the primary pathogen responsible for dental caries, frequently dominating the dental plaque microbiota of affected patients [5]. Additionally, *Lactobacillus*, *Actinomyces*, *Atopobium*, *Veillonella*, *Scardovia*, and *Propionibacterium* are closely associated with the onset and progression of dental caries [5]. Meanwhile, malnutrition has a very close relationship with the gut microbiota. Changes in the gut microbiota related to malnutrition can lead to growth retardation, inflammation and immune dysfunction [6]. At the same time, stunting and emaciation are also closely associated with dental caries [7]. The presence of dental caries can potentially impact children’s feeding behavior, thus negatively affecting their growth and development [8]. Some studies shown that children with early dental caries have higher levels of inflammatory cytokines, leading to a higher nutritional risk, which may be part of the pathogenesis of malnutrition related to dental caries [9, 10].

This study investigated the potential relationship between dental caries and malnutrition by utilizing 16 S rRNA gene sequencing and untargeted metabolomics, enhancing our understanding of the dual burden of dental caries and malnutrition in children.

## Methods

### Research design

Inclusion/exclusion criteria were applied to screen the recruited volunteers. Inclusion criteria included: (ⅰ) No obvious active bacterial or viral infections; (ⅱ) No systemic or congenital diseases; (ⅲ) No intake of antibiotics, probiotics, or other fluorides (except in toothpaste) within the past three months. Exclusion criteria included: (ⅰ) Developmental dental diseases; (ⅱ) Exogenous black dental stains; (ⅲ) Orthodontic treatment received within the past six months. All eligible volunteers were asked to collaborate with researchers in assessing their oral status and gathering dental plaque samples. Their parents were required to complete survey questionnaires (including information on anthropometric parameters and living habits) and to collect fecal samples from the volunteers. Based on the DMFT (Decayed, Missing, and Filled Teeth) index assessment, the subjects were categorized into two groups for further analysis: the caries (C) group (DMFT ≥ 1, *n* = 47) and the caries-free (CF) group (DMFT = 0, *n* = 19). Additionally, serum samples were collected from three individuals with severe dental caries (SDC group, DMFT ≥ 9) and four caries-free volunteers (CFV group, DMFT = 0). Since the subjects had mixed dentition that includes both deciduous and permanent teeth, the DMFT does not distinguish between permanent and deciduous teeth in this research.

The results of oral health assessment and information from the survey questionnaire have been presented in Table S1. Considering the problem of sample size and the fact that some habits are not closely related to caries, the some categories of habits were selected and integrated for inclusion in the analysis (Table 1).

**Table 1:** Effect of living habits on the occurrence of dental caries

Independent variables	Category of variables	*P*	OR	CI(95%)
Brushing frequency per day	-	0.004	0.017	0.001–0.278
Brushing time (compared to less than 1 min)	1–2 min	0.799	0.74	0.073–7.468
	more than 2 min	0.561	0.527	0.061–4.557
Toothbrush replacement frequency (compared to 1–3 months)	4–6 months	0.167	3.756	0.576–24.499
	7–9 months	0.6	2.687	0.067–107.937
Whether the toothpaste contains fluoride	-	0.045	0.141	0.021–0.955
Sugar intake frequency - often/always (compared to rarely/never)	-	0.03	7.793	1.215–49.967
Whether often consuming junk food	-	0.624	0.534	0.043–6.56
Taste preferences-heavy taste (compared to normal light taste)	-	0.439	2.077	0.326–13.239
Time since last dental visit (compared to no dental visit)	less than 6 months	0.956	1.077	0.078–14.903
	more than 6 months	0.073	12.5	0.793–197.094

### 16 S rRNA gene sequencing

#### Sampling

Participants were instructed not to brush their teeth for 24 h and to fast for 12 h prior to the examination and sample collection. Pooled supragingival plaque samples were collected from the buccal, lingual, and proximal surfaces of all existing teeth using oral swabs to obtain a representative profile of the supragingival microbiota. The collected samples were immediately placed in liquid nitrogen and subsequently transferred to a −80 °C freezer. To collect the fecal sample, a spoonful of sample was taken from the inside of the middle section of the fresh stool with a sterile spoon, and the fecal sample was placed in a sterile fecal DNA storage tube (CoWin Biotech, JiangSu, China). Within 24 h, the collected fecal samples were transported to the laboratory and stored in a −80 °C freezer until DNA extraction.

#### High-throughput sequencing

Total genomic DNA was extracted from dental plaque and fecal samples using the TGuide S96 DNA Extraction Kit (Tiangen Biotech (Beijing) Co., Ltd.) according to the manufacturer’s instructions. The quality of the extracted DNA were examined using electrophoresis on a 1.8% agarose gel, and DNA concentration and purity were determined with NanoDrop 2000 UV-Vis spectrophotometer (Thermo Scientific, Wilmington, USA). The hypervariable region V3-V4 of the bacterial 16S rRNA gene were amplified with primer pairs 338F: 5’- ACTCCTACGGGAGGCAGCA-3’ and 806R: 5’- GGACTACHVGGGTWTCTAAT-3’. Both the forward and reverse 16 S primers were tailed with sample-specific Illumina index sequences. The PCR products were checked on agarose gel, subsequently purified by the Omega DNA purification kit (Omega Inc., Norcross, GA, USA) and quantified using Qsep-400 (BiOptic, Inc., New Taipei City, Taiwan, ROC). After this, the amplified library was paired-end sequenced (2 × 250) on an Illumina novaseq6000. The above processes were completed by Biomarker Tech (Beijing, China).

#### Analysis

Clean reads were obtained by quality filtering, splicing paired-end sequences, merging paired sequences, and removing chimeric sequences from the original data. These clean reads were then used for feature classification to generate amplicon sequence variants (ASVs) using DADA2. Taxonomic annotation of the ASVs was performed with a confidence threshold of 70%. The alpha diversity indexes (ACE index, Chao1 index, Shannon index, and Simpson index) were calculated and displayed by the QIIME2 and R software, and the differences between groups were evaluated using Student’s *t* test. In order to assess the degree of similarity among microbial communities across different groups, the weighted UniFrac distance algorithm was employed in QIIME2 to compute the beta diversity distance matrix and its corresponding principal coordinate analysis (PCoA). Furthermore, we employed Linear Discriminant Analysis (LDA) effect size (LEfSe) to test the significant taxonomic difference among group. The impact of lifestyle variables on oral microbiota was investigated using distance-based redundancy analysis (db-RDA) with the binary Jaccard distance metric. To explore the impact of DMFT on gut microbiota differences, a redundancy analysis (RDA) was performed in R using the package ‘vegan’. Additionally, spearman rank correlation analysis was performed according to species abundance information, and correlation networks were constructed by screening data with rho > 0.1 and *P* < 0.05.

### Serum untargeted metabolomics

#### Sampling

Blood samples from both the CFV and SDC groups were collected via venipuncture from the antecubital region of the upper limb. The samples were allowed to clot at room temperature for 1 h, and then centrifuged at 2000 × g for 10 min. The resulting pale yellow supernatant serum was stored at − 80 °C.

#### Liquid Chromatography-Mass spectrometry

Serum metabolomics analysis was conducted by Biomarker Tech. A 100 µL serum sample was mixed with 500 µL of an extraction solution containing the internal standard (methanol-acetonitrile, 1:1 volume ratio; internal standard concentration of 20 mg/L), followed by vortex mixing for 30 s. The sample was then sonicated in an ice-water bath for 10 min and subsequently analyzed using an on-board assay with a 1 µL injection volume. The mobile phase for both positive and negative ion modes consisted of 0.1% formic acid in water and 0.1% formic acid in acetonitrile. Sample testing was conducted using the Waters Acquity I-Class PLUS UHPLC coupled with a Waters Xevo G2-XS QT high-resolution mass spectrometer. Primary and secondary mass spectrometry data were acquired using MassLynx V4.2 software. The raw data were processed with Progenesis QI software for peak extraction, alignment, and other data processing tasks. Compound identification was carried out using Progenesis QI software’s online METLIN database and Biomark’s proprietary library. Theoretical fragment mass deviations were maintained within 100 ppm. After normalizing the original peak area data to the total peak area, subsequent analyses were performed.

#### Analysis

The metabolites were annotated using the Kyoto Encyclopedia of Genes and Genomes (KEGG), Human Metabolome Database (HMDB), and LIPID MAPS Structure Database (LMSD). Based on the grouping situation, the fold change (FC) was calculated and compared, and the *P*-value was determined using a T-test. A modeling of the Orthogonal Projections to Latent Structures Discriminant Analysis (OPLS-DA) was performed, and its reliability was validated through permutation test. The VIP values of the metabolites were determined by multiple cross validation. Differential metabolites were screened based on the criteria of FC > 1, *P* < 0.05, and VIP > 1. The difference metabolites of KEGG pathway enrichment significance were calculated using hypergeometric distribution test.

### Data statistics

The indicators related to oral health assessment were analyzed by GraphPad Prism 8.0.2 (GraphPad Software, La Jolla, CA, USA), and the results were presented as mean ± standard deviation (SD). IBM SPSS Statistics 27 (IBM Corp., Armonk, NY, USA) was used to evaluate the effects of living habits on caries by categorical logistic regression and Chi-square test, and binary outcome disease odds ratio (OR) and 95% confidence interval (CI) were calculated. The correlations between DMFT and height-for-age Z-score (HAZ), age-specific body mass index Z score (BMI-Z) were assessed using partial correlation analysis. The significance threshold of this study was 0.05.

## Results

### Participant overview

Participants’ oral conditions and questionnaire information are presented in Table S1. The specific process of this study is shown in Fig. S1. Among the 66 qualified participants included in the analysis, the male-to-female ratio was 34:32, and the age range was 4–12 years (mean = 8.5 ± 1.7). Of the subjects, 19 (28.8%) had no dental caries and the remaining 47 (71.2%) had some degree of dental caries. The mean DMFT of caries patients was 4.40 ± 2.94. The mean plaque index (PLI) for those without caries was 0.89 ± 0.45 and 1.17 ± 0.43 for those with caries, with a significantly higher PLI for those with caries (*P* = 0.0329, mann-whitney test).

### The occurrence of dental caries is influenced by lifestyle habits and may contribute to stunting in children

The results of the binary logistic regression analysis were shown in Table 1. Brushing frequency per day and the toothpaste contains fluoride were significantly negatively associated with the occurrence of caries. Conversely, the sugar intake frequency was significantly positively associated with the incidence of caries. The 95% confidence interval for sugar intake frequency was excessively large, likely due to the small sample size. None of the other variables had a significant impact on whether the subjects developed caries.

To determine if dental caries affects the growth and development of individuals, we conducted a partial correlation analysis between DMFT and the subjects’ HAZ and BMI-Z, while controlling for three variables significantly related to dental caries. The DMFT and BMI-Z were not significantly correlated (*P* = 0.215); however, a significant negative correlation was observed between DMFT and HAZ (*P* = 0.034). This suggested that dental caries may be associated with stunting in children.

### Microbiota characteristics of patients with dental caries

#### Characteristics of the oral microbiota

The rarefaction curve tended to flatten, indicating that the sample size for sequencing was adequate (Fig. S2a). There was no significant difference in the α-diversity of the oral microbiota between the two groups (Fig. S2b-e). Additionally, the composition of the oral microbiota was similar in both groups (Fig. S2f-h). *Bacteroidota* was enriched in the CF group, while *Proteobacteria* was enriched in the C group (Fig. S2i). At the genus level, *TM7x* was enriched in the CF group, while *unclassified_Lactobacillales*, *Abiotrophia*, and *Haemophilus* were enriched in the C group (Fig. S2i). Moreover, the *Streptococcus mutans* was enriched in caries patients (Fig. S2i).

#### Characteristics of the gut microbiota

The sample size for sequencing was adequate to represent species information (Fig. S3a). There was no significant difference in α-diversity between the gut microbiota of the C group and the CF group (Fig. S3b-e). Additionally, the composition of the gut microbiota was similar in both groups (Fig. S3f-h). *Collinsella*, *Phascolarctobacterium*, *Akkermansia*, and *Catenibacterium* were enriched in the gut of caries-free individuals, while *Dialister*, *Megasphaera*, and *Agathobacter* were enriched in the gut of caries patients (Fig. S3i). Additionally, the relative abundance of Veillonellaceae was higher in patients with caries compared to those without caries (Fig. S3i).

#### Characteristics of the gut microbiota in children of different ages

To determine the influence of age-related differences in gut microbiota on the study results, we categorized the subjects into three groups for further analysis: Group A (4 ≤ age < 7, *n* = 10), Group B (7 ≤ age < 10, *n* = 37), and Group C (10 ≤ age < 13, *n* = 19). α-diversity analysis indicated that the ACE, Chao1, and Simpson indices of Group C were significantly higher than those of Group B (Fig. S4a-d). The R² value obtained from Permutational Multivariate Analysis of Variance (PERMANOVA) was very low, indicating no significant difference in gut microbial composition (Fig. S4e). The gut microbial structures of the three groups were similar, with *Firmicutes*, *Bacteroidota*, *Actinobacteriota*, and *Proteobacteria* collectively accounting for approximately 90% of the gut microbiota (Fig. S4f). At the genus level, the relative abundance of *Bacteroides* decreased with age, while the relative abundance of *Prevotella_9* increased with age (Fig. S4g). Compared to Group A, Groups B and C exhibited a lower relative abundance of *Faecalibacterium* and a higher relative abundance of *Bifidobacterium* (Fig. S4g). LEfSe analysis revealed that *Erysipelatoclostridium* and *Paraprevotella* were significantly enriched in Group A, *Holdemanella* in Group B, and *Lachnospira*, *Dorea*, and *Butyricicoccus* in Group C (Fig. S4h). Procrustes analysis showed no significant correlation between age-associated gut microbiota and caries-associated gut microbiota (Fig. S4i). The results showed that the gut microbiota significantly enriched in each age group were entirely distinct from those associated with dental caries, and no significant correlation was found between them. This suggests that the gut microbes linked to caries identified in this study are independent of the variations in age-related gut microbiota.

### Dental caries could increase the risk of stunting

To explore the potential link between dental caries and stunting, we conducted a non-targeted metabolomics analysis of serum from participants in two subgroups (the SDC group and the CFV group), while also analyzing their oral and intestinal microbiota.

#### Poor lifestyles could alter the oral microbiota

**Fig. 1:**
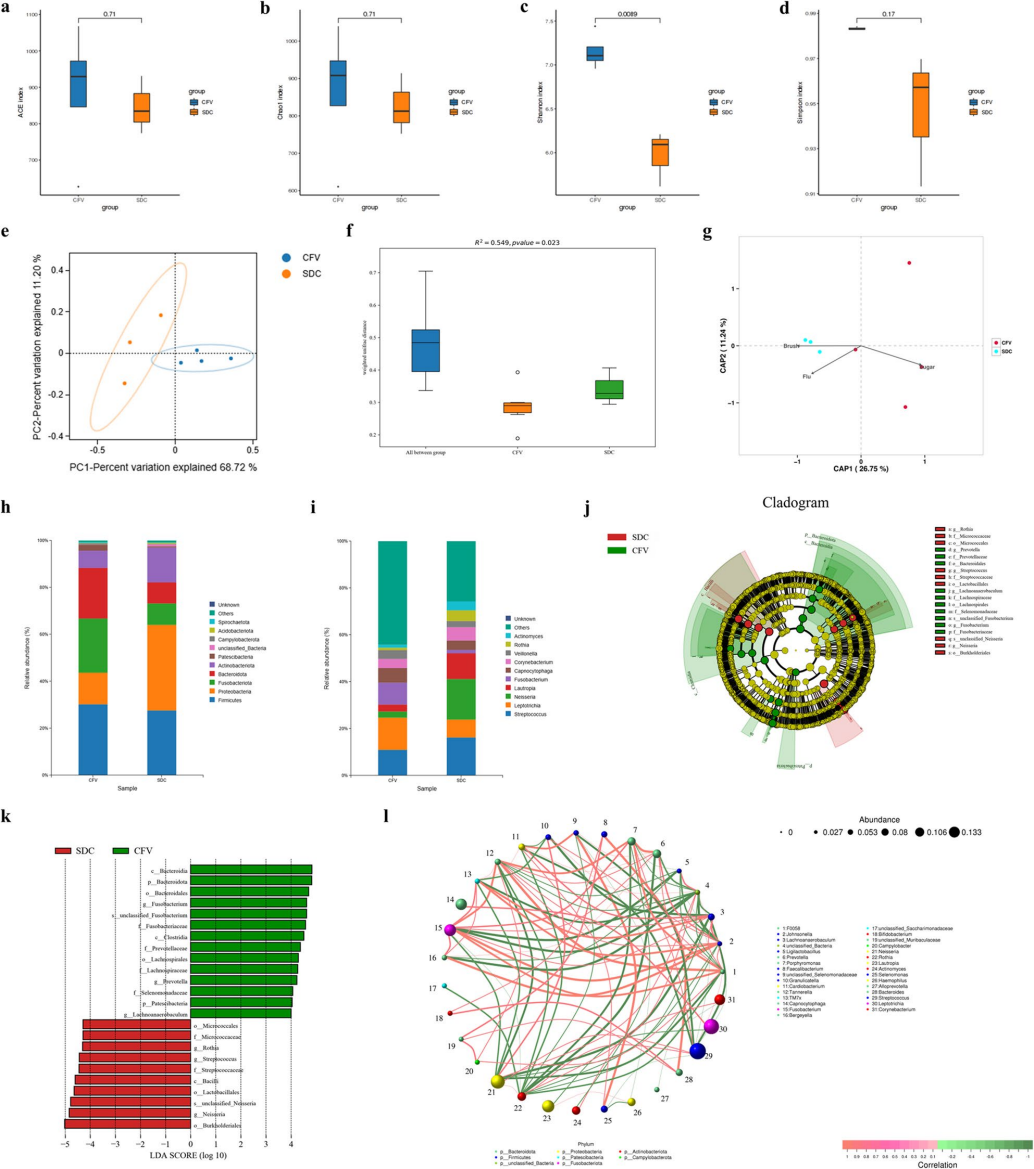
Poor lifestyles could alter the oral microbiota.** a-d.** Differences in α-diversity of oral microbiota between the CFV and SDC groups were analyzed using Student’s *t*-test. (**a**) ACE index. (**b**) Chao1 index. (**c**) Shannon index. (**d**) Simpson index. **e.** Two-dimensional PCoA plot based on the weighted UniFrac metric. The two axes represent the two feature values that contribute most significantly to the differences in microbial communities. **f.** A statistical test of β-diversity in oral microbiota between the CFV and SDC groups was conducted using the PERMANOVA, based on the weighted UniFrac metric. **g.** Evaluating the impact of lifestyle habits on oral microbiota using distance-based redundancy analysis (db-RDA) with the Binary Jaccard distance algorithm at the genus level. A longer arrow indicates a greater influence of the environmental factor on the oral microbiota; a smaller angle between the arrow and the coordinate axis signifies a stronger correlation between the environmental factor and the coordinate axis; and the closer a sample point is to the arrow, the more significant the effect of the environmental factor on that sample. "Brush" refers to the frequency of teeth brushed per day, "Flu" indicates that the toothpaste contains fluoride, and "Sugar" denotes the frequency of sugar intake. **h, i.** Bacterial composition was assessed at the (**h**) phylum and (**i**) genus levels in the oral cavity. **j, k.** Differences in the relative abundance of oral microbes, from the phylum to species level, between the CFV and SDC groups were calculated using LEfSe and presented in both (**j**) a cladogram and (**k**) a bar graph. Taxa with an LDA score greater than 4.0 are displayed in the bar graph. **l.** Spearman’s correlation network among oral microorganisms

Compared to the CFV group, species diversity was significantly lower in the SDC group (Fig. 1a-d). Additionally, a significant difference was observed in the composition of oral microbiota between the two groups (Fig. 1e, f). Among the three living habits significantly associated with dental caries, the frequency of tooth brushing had the most substantial impact on the oral microbiota of the CFV and SDC groups (Fig. 1g). The SDC group exhibited higher relative abundances of *Proteobacteria* and *Actinobacteriota*, and lower relative abundances of *Fusobacteriota* and *Bacteroidota* compared to the CFV group (Fig. 1h). The SDC group had higher relative abundances of *Streptococcus*, *Neisseria*, *Lautropia*, *Corynebacterium*, *Rothia*, and *Actinomyces*, and lower relative abundances of *Leptotrichia*, *Fusobacterium*, *Capnocytophaga*, and *Veillonella*, in contrast to the CFV group (Fig. 1i). At the family level, *Fusobacteriaceae*, *Prevotellaceae*, *Lachnospiraceae*, and *Selenomonadaceae* were enriched in the CFV group, while *Micrococcaceae* and *Streptococcaceae* were enriched in the SDC group (Fig. 1j, k). At the genus level, *Fusobacterium*, *Prevotella*, and *Lachnoanaerobaculum* were enriched in the oral cavity of the CFV group, while *Rothia*, *Streptococcus*, and *Neisseria* were enriched in the oral cavity of the SDC group (Fig. 1j, k). There were positive correlations between *Rothia*, *Streptococcus*, and *Neisseria* in the oral microbiota (Fig. 1l). The above results indicate that alterations in the oral microbiota of patients with severe dental caries may be influenced by poor lifestyle habits, such as insufficient brushing frequency, using toothpaste without fluoride, and excessive sugar intake.

#### Dental caries could cause gut microbiota dysbiosis by affecting the oral microbiota

**Fig. 2:**
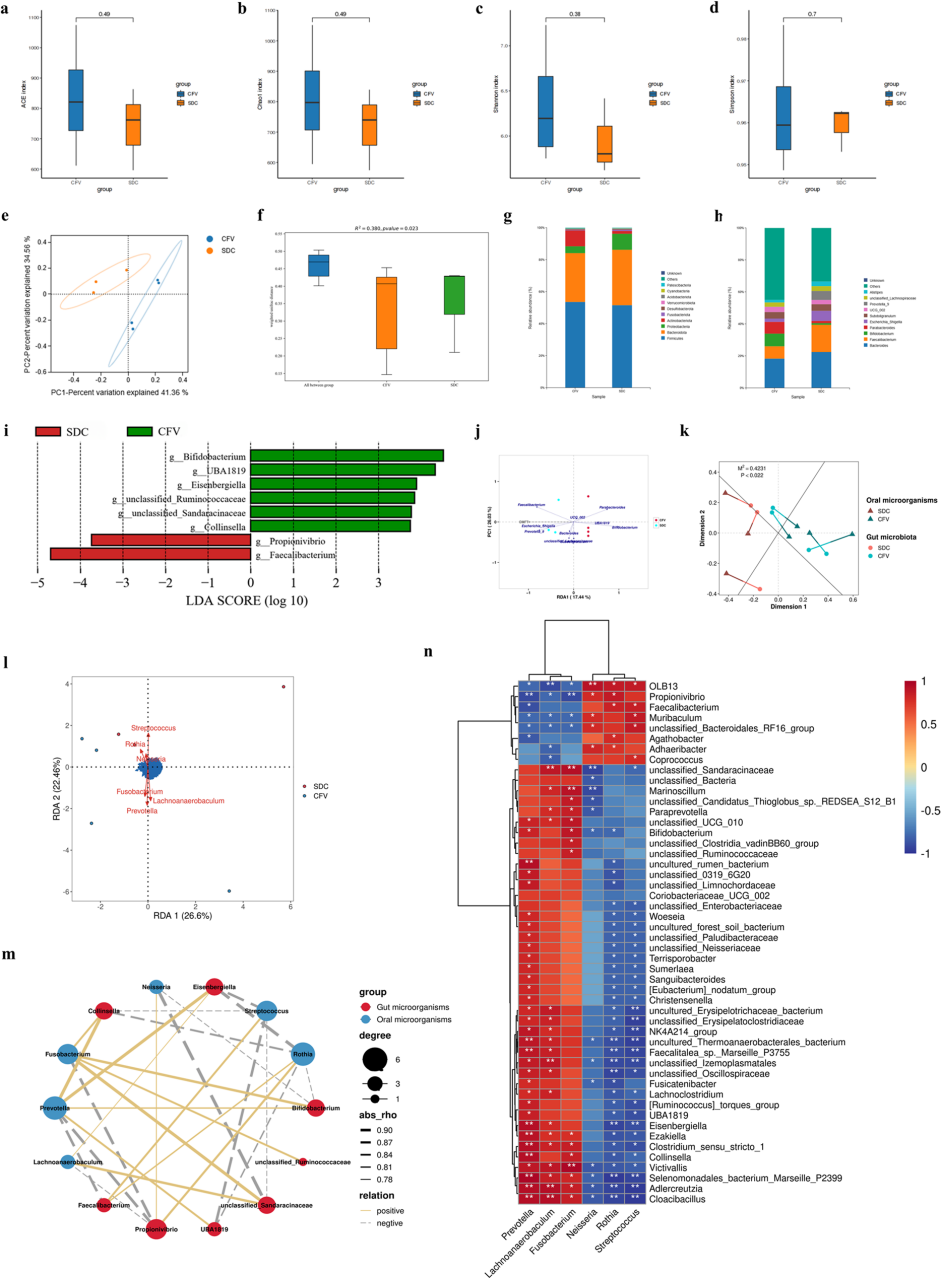
Alterations in oral microbiota associated with dental caries may lead to disturbances in gut microbiota.** a-d.** Differences in α-diversity of gut microbiota between the CFV and SDC groups were analyzed using Student’s *t*-test. (**a**) ACE index. (**b**) Chao1 index. (**c**) Shannon index. (**d**) Simpson index. **e.** A two-dimensional PCoA plot was generated based on the weighted UniFrac metric. **f.** A statistical test of β-diversity was conducted based on the weighted UniFrac distance. **g**,** h.** Bacterial composition was assessed at the (**g**) phylum and (**h**) genus levels in the gut. **i.** Differences in the relative abundance of gut microbes at the genus level between the CFV and SDC groups were calculated using LEfSe, with taxa that met an LDA score greater than 3.5 being displayed. **j.** The impact of DMFT on gut microbiota was evaluated using the redundancy analysis (RDA). **k.** The correlation between oral differential genera identified by LEfSe and the gut microbiota was assessed through Procrustes analysis based on PCoA dimensionality reduction. A smaller M^2^ value indicates a stronger consistency between the two datasets. If the *P*-value obtained from 999 permutation tests is less than 0.05, it can be concluded that the observed M^2^ value is not due to random factors. **l.** The effects of oral differential genera identified by LEfSe on the gut microbiota were evaluated using RDA. **m.** A Spearman’s correlation network was constructed at the genus level to illustrate the relationships between differential microorganisms in the oral cavity and those in the gut. **n.** A Spearman’s correlation heatmap was created for oral differential genera selected by LEfSe and gut microbes, displaying the top 50 most relevant gut microbes. * *P* < 0.05; ** *P* < 0.01; *** *P* < 0.001

The α-diversity in the gut microbiota between the CFV group and the SDC group showed no significant differences (Fig. 2a-d). The composition of the gut microbiota between the two groups exhibited a significant difference (Fig. 2e, f). In contrast to the CFV group, the SDC group had a higher relative abundance of *Proteobacteria* and a lower relative abundance of *Actinobacteriota* (Fig. 2g). In contrast to the CFV group, the SDC group exhibited higher relative abundances of *Bacteroides*, *Faecalibacterium*, *Escherichia_Shigella*, *Prevotella_*9, and *Alistipes*, and lower relative abundances of *Bifidobacterium*, *Parabacteroides*, and *UCG*_002 (Fig. 2h). At the genus level, *Bifidobacterium*, *UBA1819*, *Eisenbergiella*, *unclassified_Ruminococcaceae*, *unclassified_Sandaracinaceae*, and *Collinsella* were enriched in the gut of the CFV group, while *Propionivibrio* and *Faecalibacterium* were enriched in the gut of the SDC group (Fig. 2i).

DMFT showed a potential influence on the gut microbiota (Fig. 2j). A significant correlation was observed between the oral differential genera and the gut microbiota in both the CFV and SDC groups, with *Prevotella* and *Streptococcus* exerting the strongest influence on the gut microbiota (Fig. 2k, l). Overall, there was a significant correlation between the oral differential genera and the gut differential genera, with the exception of *unclassified_Ruminococcaceae*, which demonstrated a weaker association with the oral differential genera (Fig. 2m). The oral differential genera (*Prevotella*, *Rothia*, and *Streptococcus*) exhibited broadly significant correlations with gut genera (Fig. 2n). These results suggest that changes in the oral microbiota caused by severe dental caries can impact the gut microbiota of patients.

#### Disturbed gut microbes in children with severe dental caries might cause stunting by altering human metabolism

**Fig. 3:**
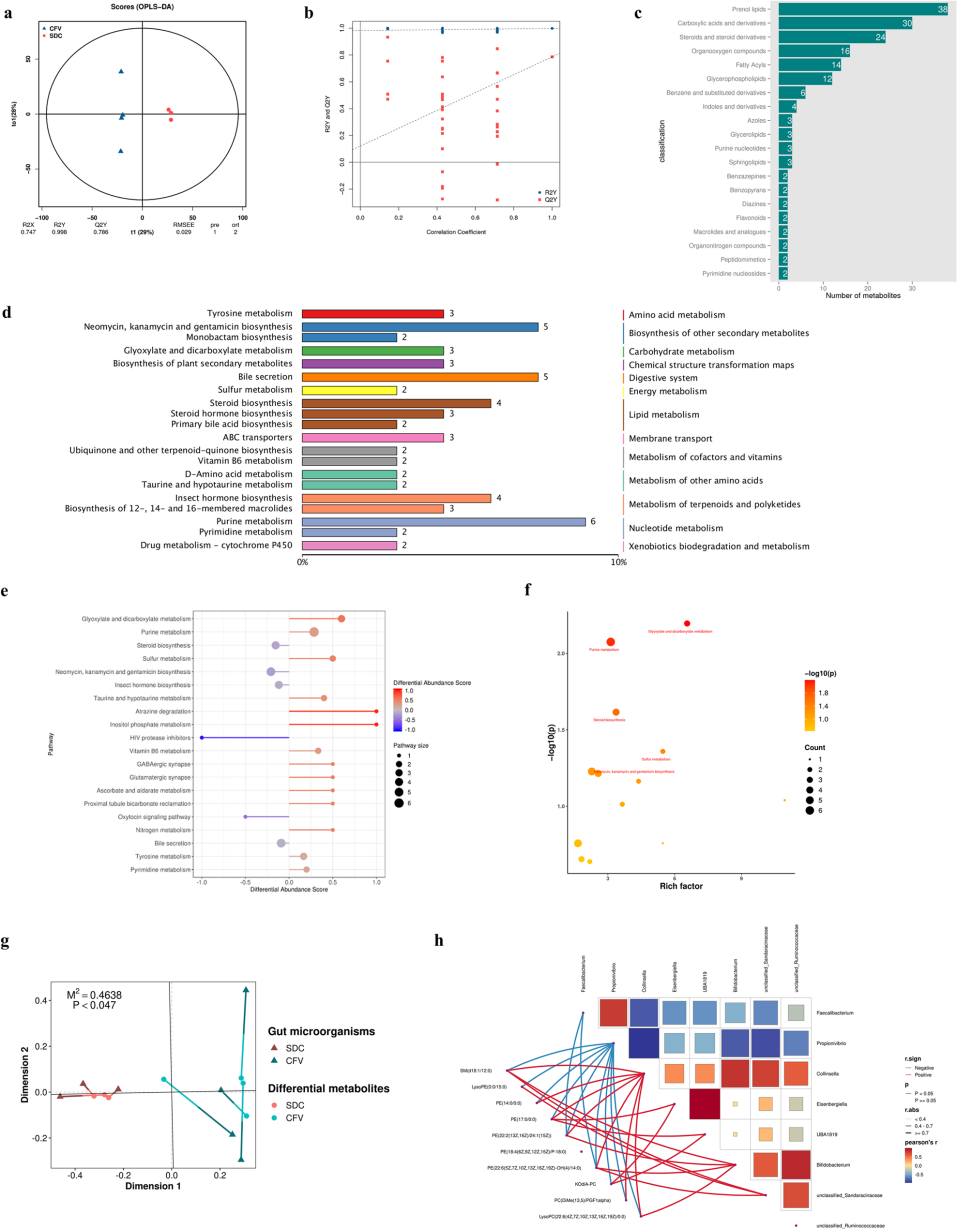
Disturbed gut microbiota associated with severe dental caries may contribute to stunting by altering metabolism**. a.** OPLS-DA. The x-axis (t1) represents the inter-group difference component, while the y-axis (to1) represents the intra-group difference component, with the percentage indicating the proportion of this component in the total variance. **b.** Replacement test of the OPLS-DA. All blue points were positioned above the red points and the slope of Q2Y fitting regression line was positive, indicating that the result of OPLS-DA was reliable. **c.** Major classes of differential metabolites. **d.** KEGG enrichment classification map of differential metabolites. The top 20 KO pathway level 3 entries with the most annotations were selected for visualization. **e.** Differential abundance score for metabolic pathway. Line segments distributed on the left side of the central axis indicate an overall downregulation trend in the pathway, while line segments on the right side suggest an overall upregulation trend. **f.** The magnitude of differences in KEGG-enriched pathways between the two groups. The *P*-values for the intergroup differences in enriched pathways were calculated using the hypergeometric distribution. **g.** Assessment of the relationship between differential gut genera and differential serum metabolites in the CFV and SDC groups using Procrustes analysis based on PCoA dimensionality reduction. **h.** Pearson’s correlation network heatmap illustrating the differential genera in the gut and the differential metabolites in serum between the CFV group and the SDC group

There is a distinct difference between the serum metabolites of the CFV group and the SDC group (Fig. 3a, b). The differences of serum metabolites between CFV group and SDC group mainly focused on prenol lipids, carboxylic acids and derivatives, steroids and steroid derivatives, organooxygen compounds, fatty acyls, and glycerophospholipids (Fig. 3c). In this study, purine metabolism, steroid biosynthesis, and bile secretion are the metabolic pathways that contain a higher number of differential metabolites (Fig. 3d). In addition, glyoxylate and dicarboxylate metabolism, sulfur metabolism, vitamin B6 secretion, taurine and hypotaurine metabolism, and purine metabolism in the SDC group were up-regulated, while steroid biosynthesis and bile secretion were down-regulated (Fig. 3e). Among these pathways, the differences in glyoxylate and dicarboxylate metabolism, purine metabolism, steroid biosynthesis, and sulfur metabolism between the two groups were statistically significant (Fig. 3f).

Procrustes analysis revealed significant associations between the differentially abundant gut genera identified by LEfSe and the serum differential metabolites in the two groups (Fig. 3g). Among the 282 metabolites that exhibited significant differences (*P* < 0.05), there were sphingomyelin (SM(d18:1/12:0)), seven phosphatidylethanolamine (PEs), three phosphatidylcholines (PCs), lysophosphatidylethanolamine (LysoPE(0:0/15:0)), and lysophosphatidylcholine (LysoPC(22:6(4Z,7Z,10Z,13Z,16Z,19Z)/0:0)). These 13 phospholipids were significantly reduced in the SDC group. These metabolites were all positively correlated with HAZ, and except for PC(O-14:1(1E)/0:0), PE(P-16:0/18:1(12Z)−2OH(9,10)), and PE(O-18:1(9Z)/0:0), the other ten metabolites demonstrated statistical significance (Table 2). *Collinsella* and *Propionivibrio* displayed the most extensive significant correlations with these ten metabolites (Fig. 3h). In contrast, the *unclassified_Ruminococcaceae*, which was least influenced by oral differential microorganisms, showed no significant associations with these metabolites (Fig. 3h). Additionally, PE (18:4(6Z,9Z,12Z,15Z)/P-18:0) did not exhibit significant correlations with the differential gut genera (Fig. 3h). In summary, the disrupted gut microbiota in patients with severe dental caries may alter serum phospholipid levels, potentially contributing to stunting in children.

**Table 2:** Correlations of differential metabolites with HAZ

Metabolites	Rho	*P*-value	Relation
PE(17:0/0:0)	0.964	0.003	positive
PE(18:4(6Z,9Z,12Z,15Z)/P-18:0)	0.964	0.003	positive
KOdiA-PC	0.929	0.007	positive
PE(14:0/0:0)	0.893	0.012	positive
LysoPC(22:6(4Z,7Z,10Z,13Z,16Z,19Z)/0:0)	0.857	0.024	positive
PC(DiMe(13,5)/PGF1alpha)	0.857	0.024	positive
SM(d18:1/12:0)	0.857	0.024	positive
LysoPE(0:0/15:0)	0.786	0.048	positive
PE(22:2(13Z,16Z)/24:1(15Z))	0.786	0.048	positive
PE(22:6(5Z,7Z,10Z,13Z,16Z,19Z)-OH(4)/14:0)	0.786	0.048	positive
PC(O-14:1(1E)/0:0)	0.750	0.066	positive
PE(P-16:0/18:1(12Z)−2OH(9,10))	0.714	0.088	positive
PE(O-18:1(9Z)/0:0)	0.429	0.354	positive

## Discussion

In this study, we found that poor living habits—such as infrequent tooth brushing, the use of non-fluoride toothpaste, and frequent sugar consumption—were closely associated with the occurrence of dental caries. Additionally, DMFT was significantly negatively correlated with the participants’ HAZ, suggesting that the development of dental caries may increase the risk of stunting in children.

It is widely recognized that the oral microbiota is directly associated with dental caries. In this study, *TM7x*, which was significantly enriched in the CF groups, is a class of bacteria with ultra-small size that parasitizes on the surface of other bacteria, lacking the capability to independently synthesize their essential nutrients [11]. The arginine deiminase system (ADS) acquired by TM7x during its colonization and evolution in the oral cavity can neutralize the acidifying effects caused by carbohydrate metabolism, thereby helping to prevent dental caries [12, 13]. It was found that in oral microbiota of C group, *Streptococcus_mutans*, *unclassified_Lactobacillales*, *Abiotrophia*, and *Haemophilus* were significantly enriched. *Streptococcus*, *Lactobacillales*, *Abiotrophia*, and *Haemophilus* have been reported to be elevated in the oral cavities of patients with dental caries or enamel lesions [14–16]. Studies have reported that the microbial diversity of dental plaque decreases as dental caries progress [17, 18], which aligns with the finding of reduced species diversity in the oral flora of the SDC group in this study. In the SDC group, *Rothia*, *Streptococcus*, and *Neisseria* were significantly enriched. *Rothia* and *Neisseria* can metabolize ethanol to produce acetaldehyde, which in turn induces oxidative stress in oral keratinocytes [19]. Almost all representative strains of *Streptococcus* are involved in the intergeneric co-aggregation of oral microorganisms [20]. *Rothia* plays a significant role in promoting microbial interactions in early dental plaque and exhibits extensive co-aggregation interactions with *Neisseria* [20]. In this study, these three genera were observed to be positively associated with one another, suggesting that they may engage in co-aggregation or cross-feeding relationships that facilitate their colonization within the biofilm. In this study, the changes in the gut microbiota of the SDC group were associated with DMFT, leading us to speculate that the oral microbiota plays an important role in this process. Subsequently, we confirmed through Procrustes analysis, RDA, and Spearman correlation analysis that the alterations in the oral microbiota of the SDC group were significantly correlated with changes in the gut microbiota. Additionally, *Dialister*, *Megasphaera*, and *Veillonellaceae* showed significant enrichments in the gut microbiota of patients with dental caries. Some studies have reported that *Dialister*, *Megasphaera*, *Veillonellaceae*, and their model genus *Veillonella* are frequently found in high abundance in the saliva of patients with dental caries [21, 22]. This suggested that they may enter the gut through saliva and influence the composition of the gut microbiota.

Changes in the serum metabolome resulting from severe dental caries may be associated with stunting in children. Glyoxylate and dicarboxylate metabolism, along with purine metabolism, play crucial roles in energy metabolism and are significantly upregulated in children with short stature [23]. Steroid hormones regulate numerous developmental and physiological processes from the fetal period through adulthood and have a significant impact on longitudinal bone growth [24, 25]. The downregulation of steroid biosynthesis and upregulation of glyoxylate and dicarboxylic acid metabolism, as well as purine metabolism, observed in the SDC group suggest a potential risk of stunting. Furthermore, We found that the differentially abundant serum metabolites between the SDC and CFV groups primarily belonged to the classes of prenol lipids, carboxylic acids and derivatives, steroids and steroid derivatives, organooxygen compounds, fatty acyls, and glycerophospholipids. These categories are associated with lipid metabolism and neural signaling pathways, playing significant roles in development [26, 27]. Prenol lipids are key components of the electron transport chain responsible for ATP generation in mitochondria and are closely linked to development, aging, and cellular bioenergetics [28, 29]. Carboxylic acids and derivatives serve as intermediates or functional molecules in human metabolism, playing essential roles in cell proliferation, organ differentiation, and system maturation [30, 31]. Steroids and steroid derivatives act as chemical messengers, secreted into the systemic circulation to influence various physiological systems, thereby regulating growth and development [32]. Organooxygen compounds encompass a wide range of metabolites (including alcohols, phenols, aldehydes, ketones, ethers, and epoxides) that are vital for bone metabolism and development, as their dysregulation is often implicated in conditions such as calcium metabolism disorders and vitamin D deficiency [26, 33, 34]. Fatty acyls represent the most fundamental lipid class in biological systems, exhibiting bioactivities that affect cellular and tissue metabolism, function, and responses to hormonal signals, including the modulation of organ development during early life [35, 36]. Glycerophospholipids are the primary structural lipids of cell membranes, mainly involved in transport, metabolic reactions, development, apoptosis, and signal transduction [37]. In this study, the levels of PC, LysoPC, PE, LysoPE, and SM in the serum of the SDC group were reduced. PC is the primary phospholipid found in mammalian cell membranes and is essential for the proliferation of human osteoblasts and bone development [38]. LysoPC produced by the hydrolysis of PC, is an important component of biomembranes involved in inflammation and bioactive phospholipids [39]. PC and LysoPC are biomarkers of malnutrition in children, playing important roles in their normal growth and development [40]. PC is typically present in bile, where it is complexed with bile salts [41]. A downregulation of bile secretion leads to a decreased amount of PC being secreted into the intestines, which impairs the absorption of fatty acids in the intestines, thereby reducing the body’s overall food intake [42]. It has been reported that mice with cholestasis exhibit decreased levels of serum LysoPC and SM [43]. SM is the predominant sphingolipid present in mammalian cell membranes, and its biosynthesis is intricately connected to PC, as the last step of SM synthesis relies on PC for the supply of the ceramide phosphorylcholine group [44]. Both PC and SM are involved in cartilage formation, which is the main determinant of linear bone growth [45]. PE can synthesize PC through the PEMT pathway and is the second largest phospholipid in mammals after PC. A longitudinal cohort study showed that children’s length-for-age Z-scores (LAZ) are positive associated with serum PC, PE, and LysoPC containing unsaturated fatty acid [46].

Gut microbiota has a profound impact on the metabolic homeostasis of the host. Serum phospholipid levels are closely related to the gut microbiota [47]. In this study, *Collinsella* and *Propionivibrio* in the gut exhibit the strongest correlation with phospholipids associated with growth and development in serum. *Propionivibrio* in the gut can ferment substrates to produce propionic acid, which influences lipid metabolism and alters the composition of phospholipid molecules in the serum [48, 49]. It has been reported that *Collinsella* can influence endogenous lipid metabolism [50]. *Collinsella* can produce lactic acid in the gut and supply fermentation substrates to bacteria that generate butyrate through cross-feeding, thereby enhancing butyrate production [51, 52]. Additionally, a study reported a *Collinsella* strain isolated from the human gut that is capable of producing butyrate [53]. The reduction of *Collinsella* in the gut of patients with severe dental caries may result in decreased butyrate levels, thereby disrupting the homeostasis of PC and SM in the serum [54]. Although existing literature supports the notion that alterations in gut microbiota associated with severe dental caries can affect serum phospholipid levels related to growth and development, the specific mechanisms underlying this phenomenon still require further investigation.

This study examines the intrinsic connections between childhood caries and stunting through the framework of the oral microbiome-gut microbiome-serum metabolome axis. This study found that dental caries can disrupt the gut microbiota by altering the oral microbiota, which subsequently modifies the serum phospholipid profile and ultimately increases the risk of adverse growth outcomes, such as stunting in children. It establishes a scientific foundation for clinical practices—such as early intervention in oral health issues to improve developmental outcomes—and advocates for the transformation of public health policies toward more integrated and preventive directions, ultimately enhancing children’s overall health. However, limitations include a small sample size and a single sampling location. Future research should prioritize multi-site, large-scale sampling to further explore this area.

## Supplementary Information

Supplementary Material 1.

## Data Availability

High-throughput sequencing data have been uploaded to the National Center for Biotechnology Information (NCBI) under accession number PRJNA1133229. The data from the untargeted metabolomics study have been deposited in OMIX at the China National Center for Bioinformation/Beijing Institute of Genomics, Chinese Academy of Sciences (Project Number: PRJCA040874).
