# Supplementary material for: The intrinsic link between the double burden of dental caries and stunting in children: the gut microbiota plays a key role
Source: BMC Microbiol. 2026 Feb 7;26:256. doi: 10.1186/s12866-026-04804-3 (PMC13001363; doi:10.1186/s12866-026-04804-3)
Supplement: Supplementary file 1 — Supplementary Material 1. [file 12866_2026_4804_MOESM1_ESM.docx]

Table S1. Human queue statistics data.

| ID | Sex | Age | Tooth decay or not | DMFT | DMFS | PLI | Height  (cm) | HAZ | Weight  (kg) | BMI  (kg·m^-2^) | BMI-Z | Brushing frequency per day | Brushing time | Toothbrush replacement frequency |
| --- | --- | --- | --- | --- | --- | --- | --- | --- | --- | --- | --- | --- | --- | --- |
| A10 | female | 6.5 | no | 0 | 0 | 1 | 120 | -0.13 | 30.0 | 20.83 | 3.61 | 2 | 1-2 min | 1-3 months |
| A12 | male | 7.0 | yes | 2 | 2 | 1 | 123 | -0.41 | 23.0 | 15.20 | -0.19 | 2 | 1-2 min | 4-6 months |
| A13 | female | 7.0 | no | 0 | 0 | 1 | 135 | 1.84 | 22.0 | 12.07 | -1.74 | 2 | more than 2 min | 1-3 months |
| A14 | male | 7.0 | yes | 1 | 1 | 1 | 128 | 0.42 | 30.5 | 18.62 | 2.01 | 2 | less than 1 min | 4-6 months |
| A15 | female | 7.0 | yes | 10 | 12 | 1 | 128 | 0.65 | 31.3 | 19.10 | 1.93 | 2 | 1-2 min | 1-3 months |
| A16 | female | 7.0 | yes | 3 | 3 | 1 | 126 | 0.32 | 26.4 | 16.63 | 0.64 | 2 | 1-2 min | 7-9 months |
| A19 | female | 6.5 | yes | 1 | 1 | 1 | 126 | 1.16 | 29.0 | 18.27 | 2.02 | 2 | 1-2 min | 1-3 months |
| A20 | male | 7.0 | no | 0 | 0 | 1 | 130 | 0.75 | 35.0 | 20.71 | 3.36 | 3 | less than 1 min | 4-6 months |
| A23 | male | 6.5 | yes | 8 | 10 | 1 | 121 | -0.21 | 22.0 | 15.03 | -0.21 | 2 | less than 1 min | 1-3 months |
| A24 | female | 6.5 | yes | 3 | 3 | 1 | 119 | -0.34 | 21.0 | 14.83 | -0.11 | 2 | 1-2 min | 4-6 months |
| A27 | female | 6.5 | no | 0 | 0 | 1 | 126 | 1.16 | 27.0 | 17.01 | 1.24 | 2 | more than 2 min | 4-6 months |
| A3 | male | 7.0 | yes | 1 | 1 | 1 | 133 | 1.26 | 37.0 | 20.92 | 3.49 | 2 | more than 2 min | 4-6 months |
| A30 | male | 6.5 | yes | 6 | 8 | 1 | 128 | 1.28 | 27.0 | 16.48 | 0.60 | 2 | 1-2 min | 4-6 months |
| A31 | male | 7.5 | yes | 2 | 2 | 1 | 124 | -0.25 | 25.0 | 16.26 | 0.40 | 2 | less than 1 min | 4-6 months |
| A4 | female | 7.0 | yes | 6 | 7 | 1 | 135 | 1.84 | 28.0 | 15.36 | -0.02 | 2 | 1-2 min | 4-6 months |
| A5 | male | 6.5 | no | 0 | 0 | 1 | 135 | 2.77 | 25.0 | 13.72 | -0.93 | 3 | 1-2 min | 4-6 months |
| A8 | male | 7.0 | yes | 3 | 3 | 1 | 120 | -0.92 | 24.3 | 16.88 | 0.89 | 2 | 1-2 min | 4-6 months |
| A9 | male | 6.5 | yes | 5 | 5 | 1 | 125 | 0.64 | 43.0 | 27.52 | 6.73 | 2 | 1-2 min | 1-3 months |
| B1 | female | 8.0 | yes | 6 | 6 | 1 | 138 | 1.39 | 24.7 | 12.97 | -1.27 | 1 | more than 2 min | 4-6 months |
| B12 | female | 7.5 | no | 0 | 0 | 0 | 130 | 0.99 | 29.0 | 17.16 | 0.82 | 3 | more than 2 min | 1-3 months |
| B15 | male | 8.0 | yes | 1 | 1 | 1 | 138 | 1.18 | 31.0 | 16.27 | 0.33 | 2 | less than 1 min | 1-3 months |
| B16 | female | 8.0 | yes | 7 | 16 | 2 | 132 | 0.43 | 24.3 | 13.95 | -0.82 | 2 | 1-2 min | 4-6 months |
| B2 | female | 8.0 | yes | 4 | 4 | 1 | 130 | 0.11 | 26.0 | 15.38 | -0.15 | 2 | 1-2 min | 4-6 months |
| B21 | male | 8.0 | no | 0 | 0 | 1 | 138 | 1.18 | 27.0 | 14.18 | -0.88 | 3 | more than 2 min | 1-3 months |
| B22 | female | 7.5 | no | 0 | 0 | 1 | 130 | 0.99 | 28.5 | 16.86 | 0.67 | 3 | more than 2 min | 1-3 months |
| B23 | male | 8.0 | yes | 5 | 5 | 1 | 136 | 0.85 | 28.0 | 15.14 | -0.32 | 2 | more than 2 min | 1-3 months |
| B26 | male | 8.0 | yes | 5 | 5 | 1 | 132 | 0.21 | 26.0 | 14.92 | -0.45 | 1 | 1-2 min | 4-6 months |
| B3 | male | 7.5 | yes | 6 | 6 | 1 | 125 | -0.08 | 23.0 | 14.72 | -0.54 | 3 | more than 2 min | 4-6 months |
| B4 | female | 7.5 | yes | 13 | 26 | 2 | 126 | 0.32 | 26.0 | 16.38 | 0.43 | 1 | less than 1 min | 4-6 months |
| C1 | male | 9.0 | no | 0 | 0 | 2 | 140 | 0.64 | 35.0 | 17.86 | 0.95 | 2 | 1-2 min | 4-6 months |
| C13 | male | 9.0 | yes | 6 | 12 | 1 | 135 | -0.12 | 29.5 | 16.19 | 0.10 | 2 | more than 2 min | 4-6 months |
| C17 | female | 9.0 | no | 0 | 0 | 1 | 138 | 0.45 | 29.0 | 15.23 | -0.36 | 3 | less than 1 min | 1-3 months |
| C18 | male | 9.0 | no | 0 | 0 | 1 | 142 | 0.95 | 40.1 | 19.89 | 1.99 | 2 | 1-2 min | 4-6 months |
| C2 | female | 9.0 | yes | 4 | 5 | 1 | 140 | 0.75 | 35.0 | 17.86 | 0.73 | 2 | 1-2 min | 4-6 months |
| C27 | male | 8.0 | yes | 6 | 8 | 2 | 130 | -0.12 | 26.0 | 15.38 | -0.18 | 2 | 1-2 min | 4-6 months |
| C37 | male | 9.5 | yes | 5 | 10 | 1 | 139 | 0.49 | 29.0 | 15.01 | -0.58 | 2 | more than 2 min | 4-6 months |
| C6 | female | 8.0 | yes | 5 | 6 | 1 | 137 | 1.23 | 30.0 | 15.98 | 0.13 | 3 | more than 2 min | 4-6 months |
| C7 | female | 9.0 | yes | 5 | 6 | 1 | 132 | -0.43 | 26.0 | 14.90 | -0.50 | 2 | more than 2 min | 1-3 months |
| C8 | female | 8.5 | no | 0 | 0 | 1 | 135 | 0.91 | 28.0 | 15.36 | -0.24 | 2 | 1-2 min | 1-3 months |
| D10 | female | 10.0 | yes | 1 | 1 | 2 | 153 | 1.60 | 47.0 | 20.08 | 1.30 | 2 | 1-2 min | 4-6 months |
| D11 | female | 9.5 | yes | 4 | 7 | 1 | 155 | 2.95 | 46.0 | 19.15 | 1.12 | 2 | 1-2 min | 7-9 months |
| D12 | male | 10.5 | no | 0 | 0 | 1 | 156 | 2.18 | 48.0 | 19.72 | 1.29 | 3 | less than 1 min | 1-3 months |
| D14 | female | 9.5 | yes | 4 | 6 | 1 | 151 | 2.37 | 36.5 | 16.01 | -0.11 | 2 | 1-2 min | 1-3 months |
| D19 | male | 10.0 | yes | 1 | 2 | 1 | 156 | 2.18 | 54.0 | 22.19 | 2.61 | 1 | less than 1 min | 4-6 months |
| D23 | male | 10.0 | no | 0 | 0 | 1 | 142 | 0.18 | 31.0 | 15.37 | -0.46 | 2 | less than 1 min | 1-3 months |
| D26 | male | 10.0 | yes | 2 | 3 | 1 | 148 | 1.04 | 33.0 | 15.07 | -0.60 | 1 | 1-2 min | 4-6 months |
| D4 | male | 10.0 | yes | 2 | 4 | 1 | 147 | 0.89 | 65.0 | 30.08 | 6.17 | 2 | 1-2 min | 1-3 months |
| D5 | female | 9.5 | yes | 4 | 8 | 0 | 141 | 0.90 | 39.0 | 19.62 | 1.30 | 2 | 1-2 min | 4-6 months |
| D9 | female | 10.0 | yes | 6 | 9 | 1 | 151 | 1.33 | 42.0 | 18.42 | 0.68 | 2 | less than 1 min | 4-6 months |
| E12 | male | 12.0 | yes | 5 | 9 | 2 | 162 | 1.11 | 65.0 | 24.77 | 2.63 | 1 | more than 2 min | 4-6 months |
| E14 | female | 11.5 | yes | 4 | 4 | 1 | 159 | 1.52 | 52.0 | 20.57 | 0.98 | 2 | more than 2 min | 1-3 months |
| E16 | male | 12.0 | yes | 1 | 1 | 1 | 160 | 0.88 | 55.0 | 21.48 | 1.44 | 2 | more than 2 min | 7-9 months |
| E17 | male | 11.5 | no | 0 | 0 | 1 | 161 | 1.92 | 65.0 | 25.08 | 2.99 | 2 | less than 1 min | 7-9 months |
| E2 | male | 11.5 | yes | 1 | 1 | 1 | 157 | 1.41 | 44.0 | 17.85 | 0.25 | 2 | more than 2 min | 7-9 months |
| E22 | male | 11.0 | yes | 2 | 4 | 1 | 147 | 0.13 | 38.0 | 17.59 | 0.28 | 2 | 1-2 min | 1-3 months |
| E4 | female | 10.5 | yes | 1 | 1 | 2 | 155 | 1.87 | 40.0 | 16.65 | -0.09 | 3 | 1-2 min | 4-6 months |
| E7 | female | 10.5 | yes | 4 | 6 | 1 | 155 | 1.87 | 35.0 | 14.57 | -0.83 | 2 | more than 2 min | 1-3 months |
| E8 | male | 11.0 | yes | 1 | 1 | 1 | 150 | 0.51 | 57.0 | 25.33 | 3.40 | 2 | less than 1 min | 1-3 months |
| E9 | female | 10.5 | yes | 4 | 7 | 1 | 148 | 0.92 | 36.0 | 16.44 | -0.17 | 1 | 1-2 min | 7-9 months |
| H1 | male | 4.5 | yes | 10 | 16 | 2 | 106 | -0.60 | 20.0 | 17.80 | 1.92 | 1 | less than 1 min | 1-3 months |
| H2 | male | 8.0 | yes | 12 | 17 | 2 | 123 | -1.25 | 24.0 | 15.86 | 0.09 | 1 | less than 1 min | 4-6 months |
| H3 | male | 4.0 | yes | 9 | 14 | 2 | 104 | -0.23 | 15.0 | 13.87 | -1.15 | 1 | 1-2 min | 7-9 months |
| N1 | female | 10.0 | no | 0 | 0 | 1 | 153 | 1.60 | 43.0 | 18.37 | 0.66 | 2 | more than 2 min | 4-6 months |
| N2 | female | 7.5 | no | 0 | 0 | 0 | 127 | 0.48 | 24.0 | 14.88 | -0.30 | 2 | more than 2 min | 4-6 months |
| N3 | female | 10.0 | no | 0 | 0 | 1 | 150 | 1.19 | 43.0 | 19.11 | 0.94 | 2 | more than 2 min | 4-6 months |
| N4 | female | 7.5 | no | 0 | 0 | 0 | 124 | -0.02 | 24.0 | 15.61 | 0.05 | 2 | more than 2 min | 4-6 months |

Table S1. Human queue statistics data (Continued).

| ID | Whether the toothpaste contains fluoride | Sugar intake frequency | Whether often consuming junk food | taste preferences | Suffering from toothache or discomfort | Time since last dental visit | There are family members who eat betel nut | Have family members smoke | Parents with oral diseases |
| --- | --- | --- | --- | --- | --- | --- | --- | --- | --- |
| A10 | no | rarely/never | yes | salty umami | yes | more than 12 months | no | no | no |
| A12 | no | often | yes | sweetness | no | more than 12 months | no | no | yes |
| A13 | yes | rarely/never | no | normal light taste | no | no dental visit | no | no | yes |
| A14 | yes | often | yes | normal light taste | no | no dental visit | no | no | no |
| A15 | no | often | yes | salty umami | yes | no dental visit | yes | yes | yes |
| A16 | no | often | yes | piquancy | no | more than 12 months | no | yes | yes |
| A19 | yes | rarely/never | yes | salty umami | no | 1-6 months | no | yes | yes |
| A20 | yes | rarely/never | no | salty umami | no | 7-12 months | no | no | yes |
| A23 | yes | always | yes | piquancy | no | 7-12 months | no | no | yes |
| A24 | yes | always | yes | piquancy | yes | more than 12 months | no | no | yes |
| A27 | yes | rarely/never | no | sweetness | no | no dental visit | no | no | yes |
| A3 | yes | always | yes | salty umami | yes | 7-12 months | no | yes | yes |
| A30 | no | always | yes | sweetness | yes | 7-12 months | no | no | yes |
| A31 | no | rarely/never | yes | normal light taste | no | more than 12 months | no | no | yes |
| A4 | yes | rarely/never | yes | sweetness | yes | 7-12 months | no | yes | yes |
| A5 | yes | often | yes | normal light taste | yes | 1-6 months | no | yes | yes |
| A8 | yes | rarely/never | yes | salty umami | yes | 1-6 months | no | yes | no |
| A9 | no | rarely/never | yes | normal light taste | no | 7-12 months | no | no | no |
| B1 | no | rarely/never | yes | sweetness | no | more than 12 months | no | no | no |
| B12 | no | rarely/never | yes | sweetness | no | more than 12 months | no | no | yes |
| B15 | yes | often | no | normal light taste | no | no dental visit | no | no | yes |
| B16 | no | rarely/never | yes | normal light taste | yes | more than 12 months | no | no | yes |
| B2 | yes | often | yes | salty umami | no | more than 12 months | no | no | no |
| B21 | no | often | yes | normal light taste | no | more than 12 months | no | no | no |
| B22 | no | always | yes | sweetness | yes | 1-6 months | no | no | no |
| B23 | no | rarely/never | yes | sweetness | no | 7-12 months | no | yes | no |
| B26 | yes | rarely/never | yes | piquancy | no | no dental visit | no | yes | yes |
| B3 | no | often | yes | sweetness | yes | more than 12 months | no | yes | yes |
| B4 | no | always | yes | sweetness | no | 7-12 months | no | no | yes |
| C1 | no | rarely/never | yes | normal light taste | no | no dental visit | yes | yes | yes |
| C13 | no | often | yes | piquancy | yes | more than 12 months | no | no | yes |
| C17 | yes | always | yes | salty umami | no | 7-12 months | no | no | no |
| C18 | yes | often | yes | normal light taste | no | no dental visit | no | yes | no |
| C2 | no | rarely/never | no | salty umami | no | no dental visit | yes | yes | yes |
| C27 | yes | always | yes | normal light taste | yes | more than 12 months | no | no | yes |
| C37 | no | often | yes | sweetness | no | no dental visit | no | no | yes |
| C6 | yes | rarely/never | yes | sweetness | yes | 7-12 months | no | yes | yes |
| C7 | yes | rarely/never | yes | sweetness | yes | more than 12 months | no | no | yes |
| C8 | yes | rarely/never | yes | salty umami | yes | more than 12 months | no | yes | no |
| D10 | no | rarely/never | no | normal light taste | yes | no dental visit | no | no | yes |
| D11 | yes | often | yes | sweetness | yes | more than 12 months | no | no | yes |
| D12 | yes | rarely/never | yes | normal light taste | no | no dental visit | no | no | no |
| D14 | no | always | yes | normal light taste | yes | 1-6 months | no | no | yes |
| D19 | no | rarely/never | yes | normal light taste | no | no dental visit | no | yes | no |
| D23 | yes | often | yes | salty umami | yes | 1-6 months | no | no | yes |
| D26 | no | rarely/never | yes | normal light taste | yes | 1-6 months | no | yes | no |
| D4 | yes | always | yes | normal light taste | yes | 7-12 months | no | no | yes |
| D5 | no | always | yes | normal light taste | yes | 1-6 months | no | no | yes |
| D9 | no | rarely/never | yes | sweetness | yes | no dental visit | no | yes | no |
| E12 | no | rarely/never | yes | normal light taste | no | 7-12 months | no | no | no |
| E14 | no | often | yes | piquancy | yes | 7-12 months | no | yes | yes |
| E16 | yes | often | yes | salty umami | yes | no dental visit | yes | yes | yes |
| E17 | yes | rarely/never | yes | sweetness | no | no dental visit | no | no | yes |
| E2 | no | rarely/never | no | salty umami | no | more than 12 months | no | no | yes |
| E22 | yes | often | yes | normal light taste | yes | 7-12 months | no | no | yes |
| E4 | no | often | yes | sweetness | no | more than 12 months | no | no | no |
| E7 | no | rarely/never | yes | salty umami | no | more than 12 months | no | no | no |
| E8 | no | rarely/never | yes | normal light taste | no | no dental visit | no | yes | yes |
| E9 | no | rarely/never | no | salty umami | yes | 1-6 months | no | no | yes |
| H1 | no | always | yes | piquancy | yes | 1-6 months | no | yes | yes |
| H2 | yes | always | yes | sweetness | yes | 7-12 months | no | yes | no |
| H3 | no | often | yes | piquancy | yes | 1-6 months | no | yes | no |
| N1 | yes | rarely/never | yes | normal light taste | no | 1-6 months | no | yes | yes |
| N2 | yes | rarely/never | yes | normal light taste | no | 1-6 months | no | yes | yes |
| N3 | yes | rarely/never | yes | sweetness | no | 1-6 months | no | yes | yes |
| N4 | yes | rarely/never | yes | salty umami | no | 1-6 months | no | yes | yes |


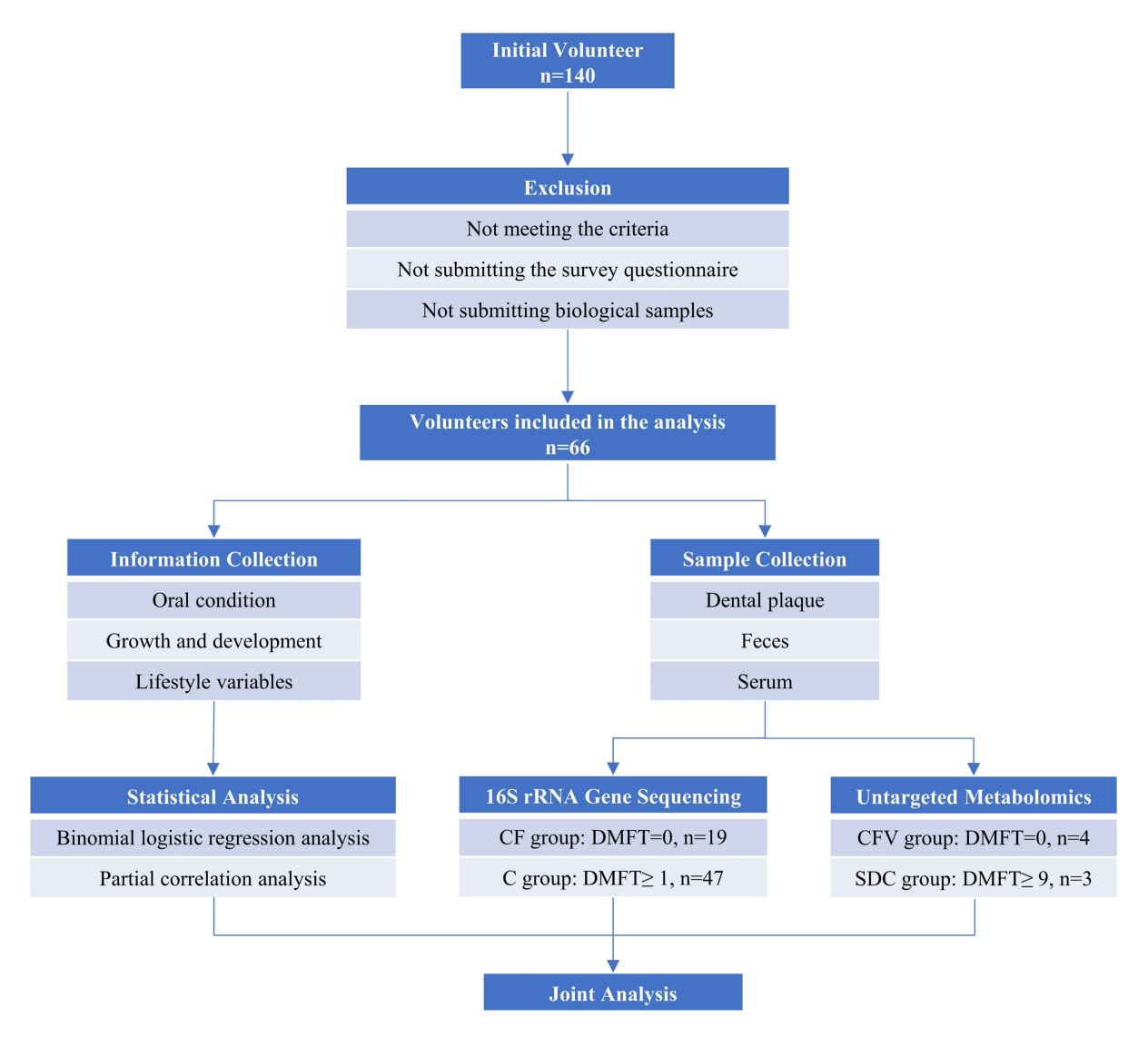


**Fig. S1. The specific process of this study.**


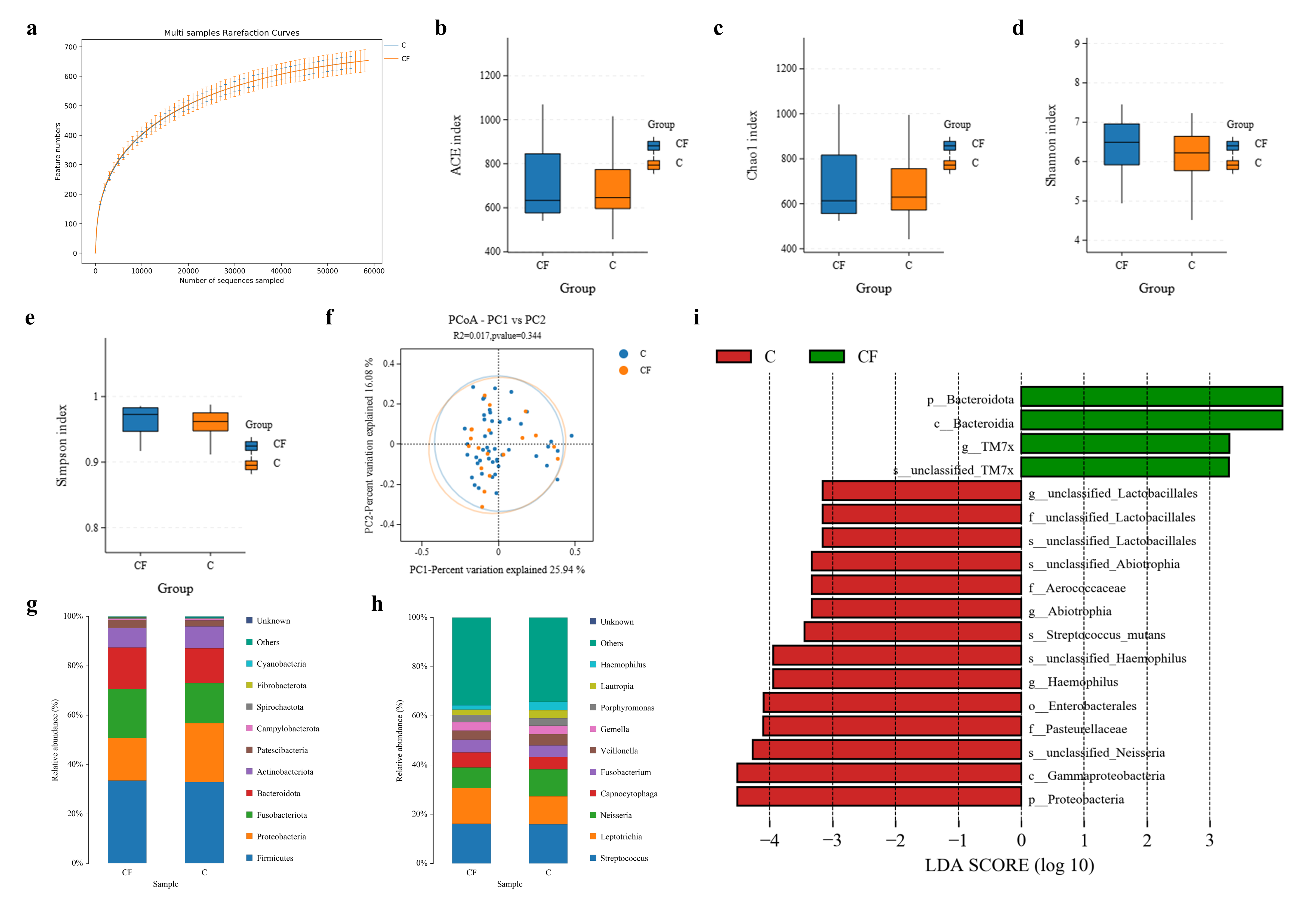


**Fig. S2. Characteristics of the oral microbiota.** **a.** Rarefaction curve of the oral microbiota. The width of the curve indicates species richness, while the smoothness of the curve reflects the uniformity of species distribution. **b-e.** Differences in α-diversity of the oral microbiota between the CF and C groups were analyzed using Student’s t-test. (**b**) ACE index. (**c**) Chao1 index. (**d**) Shannon index. (**e**) Simpson index. **f.** A two-dimensional PCoA plot was generated based on the weighted UniFrac metric, with statistical testing performed using Permutational Multivariate Analysis of Variance (PERMANOVA). **g, h.** Bacterial composition at the (**g**) phylum and (**h**) genus levels in the oral cavity. **i.** Differences in the relative abundance of oral microbes from the phylum to species level between the CF and C groups were calculated using LEfSe. Taxa with an LDA score greater than 3.0 are shown.


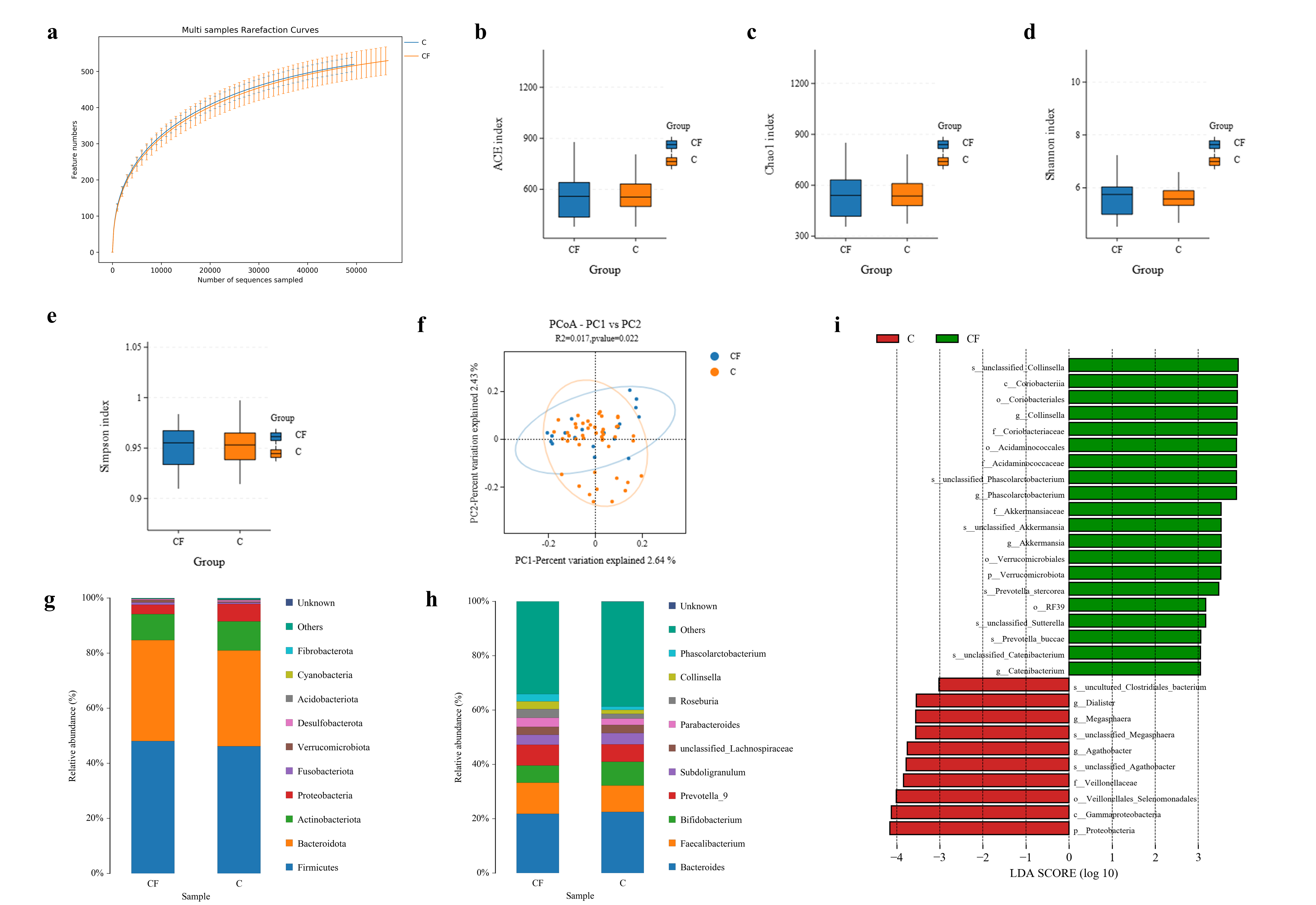


**Fig. S3. Characteristics of the gut microbiota.** **a.** Rarefaction curve of the gut microbiota. **b-e.** Differences in α-diversity of gut microbiota between the CF and C groups were analyzed using Student’s t-test. (**b**) ACE index. (**c**) Chao1 index. (**d**) Shannon index. (**e**) Simpson index. **f.** A two-dimensional PCoA plot was generated based on the weighted UniFrac metric, with statistical testing performed using PERMANOVA. **g, h.** Bacterial composition at the (**g**) phylum and (**h**) genus levels in the gut. **i.** Differences in gut microbial relative abundance from the phylum to species level between the CF and C groups were calculated using LEfSe. Taxa with an LDA score greater than 3.0 are shown.


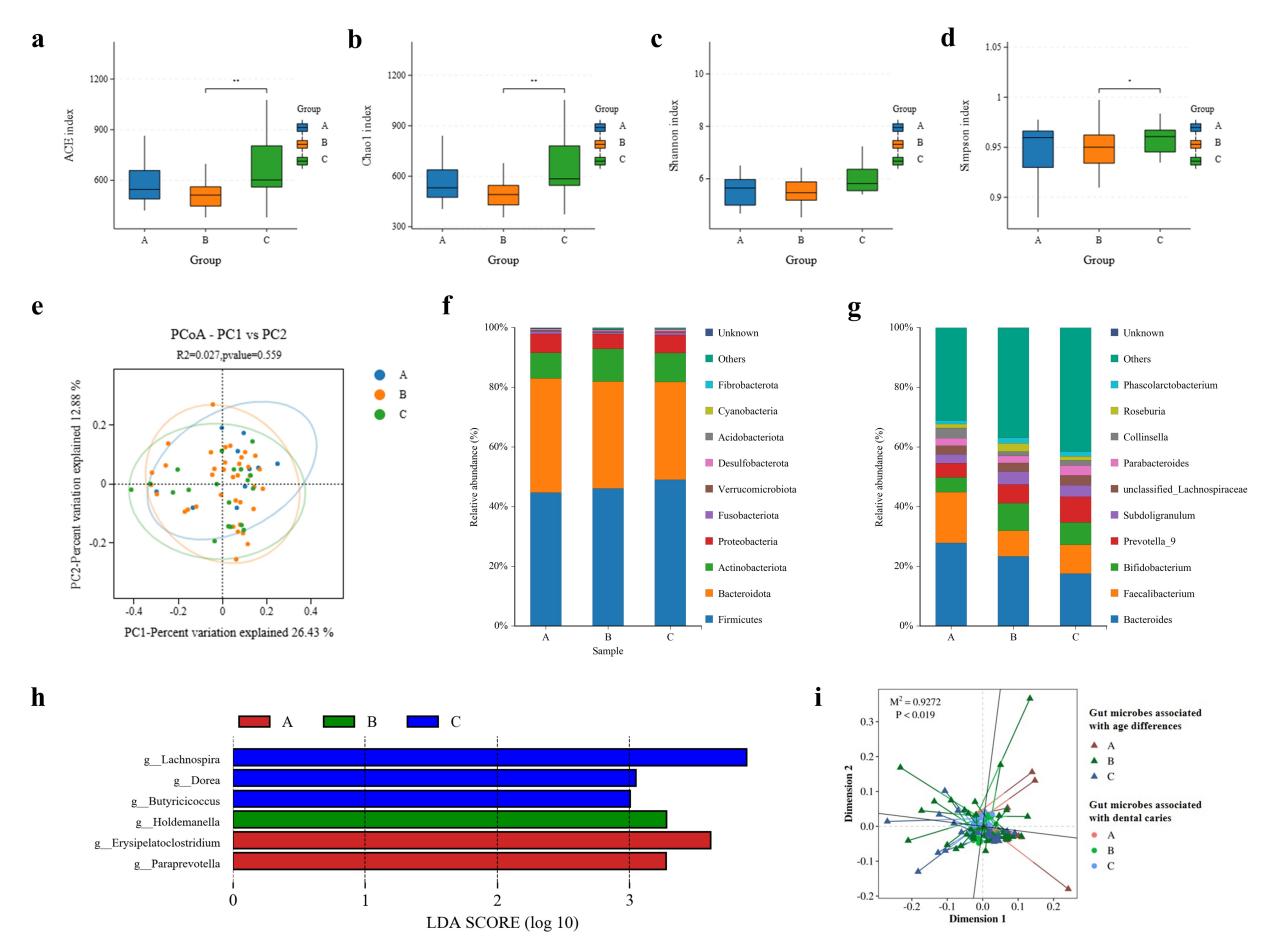


**Fig. S4. Characteristics of the gut microbiota in children of different ages.** **a-d.** Differences in α-diversity of gut microbiota were analyzed using Student’s t-test. (**a**) ACE index. (**b**) Chao1 index. (**c**) Shannon index. (**d**) Simpson index. **e.** A two-dimensional PCoA plot was generated based on the weighted UniFrac metric, with statistical testing performed using PERMANOVA. **f, g.** Bacterial composition at the (**f**) phylum and (**g**) genus levels in the gut. **h.** Differences in the relative abundance of gut microbial genera among the three groups were calculated using LEfSe. Taxa with an LDA score greater than 3.0 are shown. **i.** Using Procrustes analysis, based on PCoA dimensionality reduction, to evaluate the relationship between age-associated gut microbes and caries-associated gut microbes. * *P* < 0.05; ** *P* < 0.01.
